# Patient and Provider Perspective of Smart Wearable Technology in Diabetic Foot Ulcer Prevention: A Systematic Review

**DOI:** 10.3390/medicina57121359

**Published:** 2021-12-13

**Authors:** Giorgio Orlando, Yeliz Prior, Neil D. Reeves, Loretta Vileikyte

**Affiliations:** 1 Research Centre for Musculoskeletal Science & Sports Medicine, Department of Life Sciences, Faculty of Science and Engineering, Manchester Metropolitan University, Manchester M1 5GD, UK; n.reeves@mmu.ac.uk; 2 Centre for Health Sciences Research, School of Health Sciences, University of Salford, Salford M6 6PU, UK; y.prior@salford.ac.uk; 3 Rheumatology Outpatient Department, Mid Cheshire Hospitals NHS Foundation Trust, Leighton Hospital, Crewe CW1 4QJ, UK; 4 Diabetes, Endocrinology and Gastroenterology, School of Medical Sciences, University of Manchester, Manchester M13 9PL, UK; lvileikyte@med.miami.edu; 5 Dermatology, School of Medicine, University of Miami, Miami, FL 33146, USA

**Keywords:** diabetic foot prevention, smart wearable device, patient and provider perspectives

## Abstract

*Background and Objectives*: Smart wearable devices are effective in diabetic foot ulcer (DFU) prevention. However, factors determining their acceptance are poorly understood. This systematic review aims to examine the literature on patient and provider perspectives of smart wearable devices in DFU prevention. *Materials and Methods*: PubMed, Scopus, and Web of Science were systematically searched up to October 2021. The selected articles were assessed for methodological quality using the quality assessment tool for studies with diverse designs. *Results:* A total of five articles were identified and described. The methodological quality of the studies ranged from low to moderate. Two studies employed a quantitative study design and focused on the patient perspective, whereas three studies included a mixed, quantitative/qualitative design and explored patient or provider (podiatrist) perspectives. Four studies focused on an insole system and one included a smart sock device. The quantitative studies demonstrated that devices were comfortable, well designed and useful in preventing DFU. One mixed design study reported that patients did not intend to adopt an insole device in its current design because of malfunctions, a lack of comfort. and alert intrusiveness, despite the general perception that the device was a useful tool for foot risk monitoring. Two mixed design studies found that performance expectancy was a predictor of a podiatrist’s behavioural intention to recommend an insole device in clinical practice. Disappointing participant experiences negatively impacted the podiatrists’ intention to adopt a smart device. The need for additional refinements of the device was indicated by patients and providers before its use in this population. *Conclusions:* The current evidence about patient and provider perspectives on smart wearable technology is limited by scarce methodological quality and conflicting results. It is, thus, not possible to draw definitive conclusions regarding acceptability of these devices for the prevention of DFU in people with diabetes.

## 1. Introduction

Foot ulceration is the most devastating complication of diabetes, affecting up to one-third of people with diabetes during their lifetime [1]. It has been reported that the National Health Service in England spends over £1 billion annually managing diabetic foot ulcers (DFUs) and their consequences, which accounts for ~10% of the National Health Service diabetes budget [2]. Notably, these costs are higher than those spent for the treatment of many common types of cancer [3]. Furthermore, 20% of people with a DFU may require lower extremity amputation because of moderate to severe infection. This complication results in frequent and long-term hospitalisations and an increase in mortality at five years, which may exceed 70% in some patients [4].

Crucially, a DFU is responsible for deterioration in physical function and loss of independence in people with diabetes, resulting in psychological distress and poor quality of life [5]. Moreover, a third of people with their first DFU suffer from clinical depression. This condition is associated with an increased amputation and mortality risk compared with people with no evidence of clinical depression [6].

Although most research to date has focused on psychosocial consequences of DFUs, a growing number of studies investigate the psychological antecedents of patient engagement in preventive foot self-care [7]. Among commonly examined psychological constructs are patient cognitive and emotional representations of DFU risk [8,9,10], depression [11,12,13], personality traits [14], and cognitive functioning [10,15]. The strongest evidence so far supports the link between patient common-sense interpretation of their DFU risk and associated emotional responses, and preventive foot self-care [7,8,9,10].

Several reports showed that psychological and behavioural factors predict the first, but not recurrent DFUs [12,13]. A recent systematic review [16] reaffirmed these findings indicating that psychosocial and behavioural factors play a role in the development of first DFUs and proposed that more research is needed to determine whether these factors influence DFU recurrence. The observation that basic foot care behavioural strategies are ineffective for secondary DFU prevention was also supported by a randomised controlled trial, where the intervention group reported an improved foot self-care; however, no significant differences in DFU recurrence were observed between the control and intervention groups [17]. A subsequent study examined the role of motivational interviewing in DFU prevention and demonstrated that people with prior DFUs reulcerate despite motivational interviewing, as this group has biological DFU risk factors that are beyond control by this type of intervention [18]. The observations from descriptive and interventional studies therefore necessitate a search for more effective foot selfcare behavioural strategies, especially in secondary DFU prevention.

As we live in the era of rapidly improving medical technologies, smart wearable devices are being developed for ongoing monitoring of DFU risk factors, with the aim of DFU prevention through monitoring parameters, including foot pressure [19]. These technologies are highly effective in reducing DFU recurrence through improved foot self-monitoring [20]. Wearable devices have also been developed for monitoring wound healing and inflammatory biomarkers (i.e., cortisol, glucose, and interleukin-6), promoting them as a tool for managing diabetes and DFU [20].

Currently, intelligent socks and insoles are the main available wearable devices that show promising results in the prevention of DFU [21]. In particular, smart socks—an optical fibre-based smart textiles–allow the measurement of plantar foot temperature, whereas insole devices monitor plantar pressure through specific sensors. Both devices may alert patients via a mobile interface (e.g., smartphone or smartwatch) to modify their behaviour. Recently, a proof-of-concept study conducted in the UK demonstrated that DFU recurrence can be significantly reduced by 71% compared to the standard of care by providing timely feedback on foot pressures to individuals through smart insoles [19].

Although promising, smart wearable devices introduce additional layers of complexity in preventive foot self-care for people at high risk of DFU, and present challenges for digital literacy. Currently, information about the patient and provider perspectives on smart wearable devices and factors is limited, or facilitating their use is lacking. Several systematic reviews that have evaluated smart technology in diabetic foot disease either focused on the effectiveness of devices and not the patient experience [22], or evaluated patient experience in the management of active DFUs and not DFU prevention [23,24].

Therefore, this systematic review aimed to thoroughly examine the literature on patient and provider perspectives of usability, satisfaction barriers, and facilitators concerning the use of smart wearable devices for preventing DFU in people with diabetes.

## 2. Materials and Methods

This systematic review followed the preferred reporting items for systematic reviews and meta-analyses (PRISMA) [25]. The PROSPERO register was searched and no prior systematic review of our topic of interest was identified.

### 2.1. Search Strategy

Two reviewers (G.O. and L.V.) independently carried out the study selection, evaluation, and data extraction. The literature search was performed using three electronic databases—PubMed/MEDLINE, Scopus, and Web of Science—from 22 January 1984 to 10 October 2021. An examination of the reference lists of included studies were also conducted to identify other potentially relevant works. Rayyan software [26] was used to screen and select the articles as well as exclude duplicates.

The following search terms were used: “diabetes mellitus” OR “diabetic foot” OR “diabetic foot ulcer” OR “diabetic foot prevention” AND “digital technology” OR “smart technology” OR “smart device” OR “smart wearable device” OR “intelligent insoles” OR “smart insoles” OR “smart socks” OR “smart shoes”. The search terms were adapted for use in the different databases.

### 2.2. Study Selection

Inclusion criteria for the selection of appropriate studies were defined as follows: population: people with a diagnosis of type 1 or type 2 diabetes mellitus and at high risk of developing a DFU; intervention: studies investigating the role of smart wearable technology in DFU prevention; outcome measure: patient or provider perspectives on usability, satisfaction, barriers, and facilitators concerning smart wearable devices; study design: Quantitative, qualitative, or mixed studies.

Articles examining people with an active DFU, written in languages other than English, or not published in international peer-reviewed journals were excluded. Furthermore, articles exploring nonwearable devices aimed at DFU prevention, wearable devices aimed at monitoring active DFU, or focused on treatment of DFUs were also excluded. Commentaries and review articles were not considered. Disagreements between reviewers on the eligibility of the articles were discussed, and a final decision was made based upon the consensus of all authors.

### 2.3. Data Extraction and Collection Process

Two authors (G.O. and L.V.) independently read the selected articles and recorded and extracted data using a structured proforma on Excel. This proforma included the origin and year of publication, demographic data (sample size, sex, and age), clinical information (type of diabetes, presence of chronic complications, and history of DFU), methodology (study design, device type, quantitative and qualitative tools), outcomes, primary findings, and general conclusions.

### 2.4. Methodological Quality Evaluation

The methodological quality of the studies included in this review was ascertained by G.O. and L.V. The quality assessment tool for studies with diverse designs (QATSDD), a 16-item scale appropriate for qualitative, quantitative, and mixed methodologies, was used for this purpose. The validity and reliability of QATSDD have previously been established [27]. A score ranging from 0 to 3 (0: not at all; 1: very slightly; 2: moderately; and 3: complete) was given for each item. Total scores were translated into percentages and the score for each item was reported as the mean. Any discrepancies among reviewers were resolved through an iterative consensus approach.

## 3. Results

### 3.1. Study Characteristics

**Figure 1:**
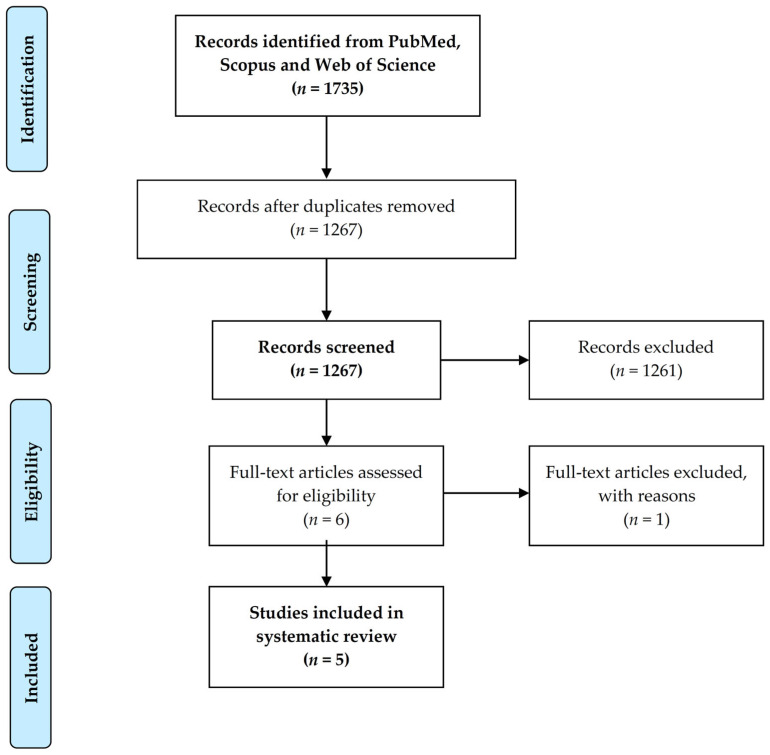
PRISMA flowchart illustrating the process of study selection.

Figure 1 shows the PRISMA flow diagram. The literature search revealed a total of 1735 articles, of which only 6 were considered eligible for inclusion. Of these, one article was excluded after thorough examination, as it did not meet the inclusion criteria [28]. Thus, a total of five articles were included in the analysis [29,30,31,32,33]. The publication year of these studies ranged from 2017 to 2021. Three articles used a mixed quantitative/qualitative design [31,32,33] and two were quantitative in nature [29,30].

The characteristics of the studies included are summarized in Table 1. Three of the five studies (60%) were patient-related [29,30,31], one focused on the healthcare provider [33], and one included both patient and provider perspectives [32]. Four articles (80%) considered the use of smart insole devices [29,31,32,33] and one article focused on smart socks [30].

Four reports [29,31,32,33] used theory-based assessment tools to evaluate patient and provider perceptions of usability and ease of use, whereas one study [30] employed an in-house-designed questionnaire to examine patient levels of satisfaction with the device. The heterogeneous nature of the study designs, aims, and key findings precluded a meta-analysis of the data.

### 3.2. Results of the Methodological Quality Assessment

The methodological quality rating of the articles, expressed as a percentage of the total possible score (100%), ranged from 31 to 76.2%, with a mean score of 59.6% for all the papers. The quantitative studies obtained a lower total score compared with their qualitative counterparts (QNT: 42.9% vs. QLT: 69.8%). These scores indicate a risk of bias ranging from medium to high.

Table 2 shows the means of the 16 methodological criteria according to the study design. Significant criticisms, with scores lower than 1, were reported in the quantitative studies for the items exploring sample size, recruitment procedure, validity, and reliability of the tools implemented and user involvement in the study design. A score of 0 was found for user involvement in the study design. Higher scores (range: 2 to 2.25 points) were reported for the criteria concerning the description of the aims/objectives and research setting, as well as the rationale for the choice of the data collection tools. The remaining items obtained a score ranging from 1.25 to 1.75 points.

The scores for most of the items (11/14) were higher for the qualitative studies when compared with the quantitative articles. The highest scores (range: 2 to 3 points) were reported for the following criteria: theoretical framework, aims/objectives, data collection procedure, rationale for the selected data collection tools, assessment of reliability of analytic process, appropriateness of data collection methods, and methods of analysis used to answer the research questions. Lower scores (approximatively 1.5 points) were noted for the items concerning sample size, descriptions of the research setting and recruitment, assessment of the data collection tool for reliability or validity (quantitative tools), and the discussion of the strengths and limitations. Finally, a score of 0 was reported for user involvement in the study design.

### 3.3. Patient Perspective

Three articles included a total of 62 patients with and without a history of DFUs [29,30,32], whereas one article only defined the presence of a previous history of DFU in a cohort of 53 diabetic patients [31]. Among these, Reyzelman et al. [30] showed that patients are open to using sock devices, as they appear safe, comfortable, well-designed, and potentially useful in preventing DFU. They also stated that the mobile application was easy to use. A high level of satisfaction was reported with this device. Similarly, Najafi et al. [29] revealed that wearing smart insoles over three months was a pleasant experience for patients and that the device was considered easy to use and effective by the patients. Again, a high level of satisfaction was noted for this device.

Another study examined the psychosocial factors explaining the intention of patients to use smart insole devices [31]. It reported that self-efficacy (i.e., the belief that one could develop the skills to use a smart insole) and attitude (i.e., positive or negative feelings towards using the smart insole) were the strongest independent predictors of behavioural intention. Effort expectancy (or ease of use) and performance expectancy (i.e., the belief that smart insoles will prevent DFUs) were moderating factors. These quantitative findings were further substantiated by a thematic analysis of a focus group discussion conducted with people at high DFU risk. The results revealed that attitude, self-efficacy, performance expectancy, and effort expectancy combine to predict the intention to adopt smart insole technology.

In a subsequent study, the same group of researchers evaluated the feasibility of podiatrist-led health coaching to facilitate smart shoe insole adoption in persons at high DFU risk [32]. After 4 weeks of intervention, the unified theory of acceptance and use of technology (UTAUT) questionnaire scores decreased significantly, particularly in the performance expectancy, attitude, and behavioural intention domains. The qualitative findings demonstrated that although participants could appreciate the potential benefits of the smart insole, they did not intend to adopt its current version in the future. They reported frustration when the device malfunctioned and felt that repeated alerts were becoming intrusive during daily activities. For some participants, especially those who had not previously experienced DFUs, the feedback appeared random and significantly diminished their level of trust in the device. On the other hand, those with previous DFUs, even though they believed the device was providing accurate feedback, felt that there was little they could do to manage high pressure areas under the feet. Furthermore, they reported that comfort was an issue because of the restrictiveness caused by the need to wear lace-up or Velcro-enclosed footwear. Patients concluded that several improvements were needed before the device could be used in the diabetic population.

### 3.4. Provider Perspectives

Together, the two studies on healthcare providers included 121 private and public podiatrists. Macdonald et al. showed that, among the podiatrists, performance expectancy was the single most important predictor of behavioural intention to adopt a smart insole by a clinical practice [33]. Qualitative analyses indicated that podiatrists believed that wearable technology would improve foot self-management. They also indicated that smart insoles would be best for those with previous DFUs, as these people would be more motivated to wear shoes that accommodate the device. However, concerns were raised about cost, footwear and the device’s functionality with elderly and remote populations [33].

A more recent survey indicated that podiatrists’ attitudes towards smart insoles were strongly influenced by their patients’ views. Disappointing participant experiences with smart insoles negatively impacted podiatrists’ intentions to adopt the device into practice. However, study podiatrists still saw value in real-time foot monitoring and indicated that device refinement would increase the likelihood of future adoption [32].

## 4. Discussion and Conclusions

This systematic review is the first to explore patient and provider perspectives on the acceptance of smart wearable devices in people at high risk of DFU, and to examine the factors limiting or facilitating their use in clinical practice. We identified only a limited number of quantitative and qualitative articles (five studies), which were at moderate-high risk of bias, with quantitative studies rating lower on methodology scores than qualitative studies. The main methodological issues were inadequate sample size, poorly defined recruitment procedures, the lack of psychometric description of the assessment tools, and in particular, the absence of user involvement in the study design. Moreover, the results of these studies were partially conflicting. For example, although the report by Najafi et al. [29] demonstrated that the study participants receiving more alerts in response to elevated plantar pressure adhered better to wearing the device and reported greater satisfaction with the device, Macdonald et al. [32] showed diminished patient responsiveness to alerts and general fatigue with using the device over time. Current evidence is therefore inconsistent, and it is not possible to draw definitive conclusions about patient and provider perspectives on the usability of these devices for the prevention of DFU. Furthermore, only one group of researchers [31,32,33] has explicitly addressed psychological factors influencing patient and podiatrist acceptance of smart wearables. There were differences among patients and podiatrists in factors that influenced their behavioural intention to adopt a smart insole: although attitudes towards smart technology and self-efficacy were key in activating patients, the performance expectancy was a single most important factor motivating podiatrists to adopt smart insoles in their clinical practice. The qualitative findings [32] clarified, at least to some extent, the somewhat unexpected trend towards a significant reduction in smart insole performance expectancy scores: the performance of the device used in this study did not meet participants’ initial expectations. As a result, despite participants’ initial adoption of the smart insole, their attitude and behavioural intention towards future adoption were negatively impacted by their experiences.

These observations highlight an important limitation of the studies that use behavioural intention as a proxy for technology acceptance, thereby providing little insight on actual technology use. Moreover, they do not account for health system complexity and temporality, which require an approach to smart technology adoption as a dynamic and interactive system. This necessitates that as the relationship between people at high risk for DFU and technologies develop, technology implementation is evaluated longitudinally so that emerging issues between people at high DFU risk and care delivery processes can be identified and addressed. Thus, there is a need for timely patient and provider involvement in the development and delivery of such technologies if we are to promote sustained behaviour change. Furthermore, the theoretical models guiding such studies are typically social cognition models and thus do not incorporate DFU-specific domains, such as patient perceptions of their DFU risk or specific emotional responses that were previously identified as important predictors of preventive foot self-care [7].

Thus, adequately powered research is needed to assess longitudinally whether the patient and provider factors identified by these studies are effective in activating people at high DFU risk and in preventing diabetic foot ulceration.

## Figures and Tables

**Table 1:** Summary of studies exploring patient and provider perspectives on the usability of smart wearable devices in people at high risk of diabetic foot ulcer.

Reference	Study Design	Participants	Clinical Condition	Sample Size	Type of Device	Data Collection Tools	Main Findings
Najafi et al. [29]	Quantitative	Patients	DPN	17	Insoles	Questionnaires	The device and smartwatch were considered easy to use and effective by the patients. A high level of satisfaction was reported. More frequent pressure alerts were associated with better adherence, improvement in offloading, and greater satisfaction with the device.
Reyzelman et al. [30]	Quantitative	Patients	DPN with and without a history of DFU	35	Socks	Questionnaires	Patients described the socks as useful, comfortable, well-designed, and easy to wear. The mobile application was easy to use. Patients were highly satisfied with the device and mobile application.
Macdonald et al. [31]	Mixed	Patients	Diabetic patients with and without a history of DFU	53	Insoles	Questionnaires/focus groups	Attitude, self-efficacy, performance expectancy, and effort expectancy were predictors of the patients’ behavioural intention to use an insole system.
Macdonald et al. [33]	Mixed	Podiatrists	-	111	Insoles	Questionnaires/focus groups	Podiatrists considered the insole device as a useful tool for monitoring diabetic foot disease. Performance expectancy was the main predictor of the intention to use the device in clinical practice. Providers raised several concerns about the cost, type of footwear, and functionality of the insole device with elderly and remote populations.
Macdonald et al. [32]	Mixed	Patients/Podiatrists	DPN	10/2	Insoles	Questionnaires/focus groups	Patient perspective: Performance expectancy, attitude, and behaviour intention decreased significantly after 4 weeks of intervention. The patients were particularly concerned about alert intrusiveness and restricted choice of footwear. Although they appreciated the potential benefits of the smart insole, they did not intend to adopt its current version in the future. Provider perspective: Patients’ negative experiences with the insole device negatively impacted the podiatrists’ view of the device. Although the device was regarded as being useful for foot monitoring, podiatrists were of the opinion that its performance and usability require several improvements.

Abbreviations: DFU: diabetic foot ulcer; DPN: diabetic peripheral neuropathy.

**Table 2:** Summary of methodological quality scores according to the study design.

Criteria (Score: 0 to 3 Points)	Quantitative Studies [29,30] (Mean Values)	Mixed Studies [31,32,33] (Mean Values)
Explicit theoretical framework	1.5	3
Statement of aims/objectives in main body of report	2.25	2.7
Clear description of research setting	2.25	1.5
Evidence of sample size considered in terms of analysis	0.25	1.3
Representative sample of target group of a reasonable size	0.75	1.3
Description of procedure for data collection	1.75	2.5
Rationale for choice of data collection tool(s)	2	3
Detailed recruitment data	0.75	1.3
Statistical assessment of reliability and validity of measurement tool(s) (Quantitative only)	0.75	1.3
Fit between stated research question and method of data collection (Quantitative only)	1.75	2.7
Fit between stated research question and format and content of data collection tool e.g., interview schedule (Qualitative only)	-	2.5
Fit between research question and method of analysis (Quantitative only)	1.5	3
Good justification for analytic method selected	1.25	2
Assessment of reliability of analytic process (Qualitative only)	-	2.5
Evidence of user involvement in design	0	0
Strengths and limitations critically discussed	1.5	1.5

## Data Availability

Not applicable.
